# Arseniate of Soda in Scrofula

**Published:** 1861-08

**Authors:** 


### Selections.

### ARSENIATE OF SODA IN SCROFULA.

By Dr. BOUCHUT.

*From Braithwaite's Retrospect.*

The manifestations of the scrofulous diathesis are all exceedingly inveterate, and the treatment directed for their cure is only too often unsuccessful. All forms of scrofula, however, are not equally obstinate. The tertiary forms, which appear as tuberculosis, are generally incurable; of the other forms, whether primary or secondary, those which affect the bones are especially tenacious and of long duration; and, in truth, it is only over the mucous, glandular, and cutaneous manifestations of scrofula that therapeutics can exert any decided power. The author has used in turn all the medicines usually reputed anti-scrofulous: iodine, mercury, iron, baryta, bromine, extract of walnut leaves, cod-oil, arsenic, etc.; but, of all these agents, the arseniate of soda has appeared to him to be, under the circumstances which he describes, the most energetic and the most efficacious. In suitable doses, arsenic is one of the best *tonics* and *corroborants* we possess; it is only in too large doses, or when its use is too long continued, that it deserves to figure as an *alterative.* As a tonic, it is an admirable succedaneum of iron, quinine, and cod-liver oil; and is of great service in the majority of organic and nervous cachexiæ. In the scrofulous cachexia it is an excellent remedy; and under its influence children ordinarily regain their appetite, strength, and colour. In such cases there is amelioration only; but the cases where it cures are those in which the diathesis has not yet produced a cachectic condition, and where the local manifestations are superficial and confined to the skin, the mucous membrane, and suppurated lymphatic glands. Beyond these, in tuberculosis and the diseases of bones, it is only a good palliative. But, although thus limited, the therapeutic effects have considerable importance. It is no slight matter to be able to abridge the duration of a coryza, an ophthalmia, or the suppuration of glands, cutaneous ulcers, otorrhœas, leucorrhœas, etc., which depend upon the scrofulous constitution. The author possesses a number of facts relating to such cases, as well as to scrofulous perforation of the palate, seputed syphilitic, suppurating cervical glands (formerly called *rcrouelles*), etc. The result in all of them was the same; a rapid cicatrization of the sores was always observed. The following are some examples:

*Obs.* I. A female child, *aged* 10, entered the Hospital Ste. Eugénie on 3d June, 1858; born of scrofulous parents; pale, thin, and weak, with an open scrofulous sore under the chin, discharging an abundant suppuration; below the angle of the jaw, other enlarged glands, not suppurated; appetite and digestion natural. Arseniate of soda was prescribed in increasing doses, from 5, 10, 15, to 20 milligrames (1-14 to 1-4th grain) in a julep. Taken from July to December, the medicine produced no disturbance in the digestive organs. On October 15th, the child was completely cured, and was dismissed on December 27th. The tumors of the neck had disappeared, and there remained a solid cicatrix of good appearance under the chin.

*Obs.* II. Girl of 13; two large scrofulous ulcers in the neck, with abundant suppuration, and the skin undermined. Arsenate of soda in increasing doses. Cure in three months.

*Obs.* III. A girl of 10, suffering from osteitis of the phalanges of the big toe, onyxis, and suppuration of the matrix of the nail; treated with arseniate of soda internally, and foot-baths of chlorate of potash. In three months the wound had completely healed, although there still remained some swelling of the toe, and the skin was red and tender. The child had colour, had a keen appetite, and became stout and plump.

The fourth case, a girl of 11, affected with lichen, and presenting numerous glandular enlargements and fistulous ulcers under the jaws, had a chronic abscess of the chest, which had been punctured and injected with iodine. She had been treated with cod-liver oil for three months, but had lost flesh, and the abscesses continued to discharge an abundant suppuration of an unhealthy character. On May 15th, the arseniate of soda was commenced. On July 15th, the abscesses were almost cured; and the child had a fresh healthy colour, and had gained flesh. On September 1st, the abscesses on the neck and chest were completely closed. The cure continuing, the child was dismissed, November 8th. Since that date all the scrofulous children presenting superficial cutaneous affections in M. Bouchut's wards have been treated in the same way, and the result has always been favourable.

In the case of a girl, aged 11, affected with swelled submaxillary glands, and large scrofulous ulcers extending from the ear downwards under the chin on both sides, the improvement began after a fortnight's treatment with the arseniate of soda, and she left the hospital cured in four months.

Another girl, the subject of tinea tonsurans and osteitis of the big toe had been ill for a year, and entered the hospital, July 8th, 1858. The general health was pretty good; with the exception of some paleness, there was no functional disorder. Epilation, lotions of corrosive sublimate, applied to the head; arseniate of soda internally; sulphurous baths. In November, 1859, the sore of the foot had completely cicatrized; but the tinea tonsurans had spread. The epilation was continued, and she was dismissed cured on the 15th October, 1860.

In all these cases the arseniate of soda was begun in doses of 5 milligrammes (1-14th grain), and increased at the end of a few days to 10,15 and finally 20 milligrammes (1-4 grain.) Beyond that dose, symptoms of gastralgia, vomiting and diarrhœa are apt to come on, and should be guarded against. It may be given in gum julip, in Bordeaux wine, in syrup of cinchona, or syrup of gum. The following formula may be employed, and the medicine left in charge of families, for use during several weeks: Syrup of cinchona, 300 grammes (ʒ x)*; arseniate of soda, 5 centigrammes (gr. j.); one or two teaspoonfuls each day; each teaspoonful containing about 1 milligramme (1-70th grain) of the arseniate of soda. In this dose, and with the precaution of increasing it gradually, the arseniate of soda presents no danger. Its effects are, to increase the appetite and produce a richer sanguification, manifested by a ruddy colour of the skin, muscular energy, and an unmistakable appearance of health. Such results, says the author, are not to be despised in the case of scrofulous subjects, pale, emaciated, and exhausted by long suppurations and mucous discharges; and it is on

* This should, evidently, be ℥'s, instead of ʒ's.

these grounds that he recommends the arseniate. He does not propose it, however, as a specific, but only as a tonic or corroborant, which stimulates the appetite and imparts increased activity to the molecular nutrition of the tissues. In scrofulous constitutions, it is the slowness of the movements of nutrition, and of the exchange of the circulating materials, which gives the diseases their peculiar character. In this respect the arsenical medication is useful, as perhaps the cod-liver oil is also, by stimulating nutrition; and the results obtained should induce practitioners to have resource to it. It must be noted, however, that the arseniate of soda is suitable only in scrofulous diseases of the cutaneous, mucous, and glandular textures. Its efficacy is doubtful in diseases of the bones; and it is only a palliative in the case of tertiary scrofula, that is in tuberculization.—*Bull. Gen. de Therapeutique.—Edinburgh Medical Journal, March,* 1861, *p.* 830.

